# Lower eyelid angioleiomyoma: a rare case of a slowly progressive orbital tumor with a 10-year history

**DOI:** 10.1093/jscr/rjaf507

**Published:** 2025-07-14

**Authors:** Amine Oussalem, Anas Azgaoui, Bouchra Dani, Malik Boulaadas

**Affiliations:** Speciality Hospital, Department of Maxillo-Facial Surgery, Area Lamfadel Cherkaoui, Rabat - Institut, Rabat B.P 6527, Morocco; Speciality Hospital, Department of Maxillo-Facial Surgery, Area Lamfadel Cherkaoui, Rabat - Institut, Rabat B.P 6527, Morocco; Speciality Hospital, Department of Maxillo-Facial Surgery, Area Lamfadel Cherkaoui, Rabat - Institut, Rabat B.P 6527, Morocco; Speciality Hospital, Department of Maxillo-Facial Surgery, Area Lamfadel Cherkaoui, Rabat - Institut, Rabat B.P 6527, Morocco

**Keywords:** angioleiomyoma, eyelid tumor, orbital mass, smooth muscle tumor, histopathology

## Abstract

Angioleiomyoma is a rare benign vascular smooth muscle tumor, exceptionally reported in the orbit and peri-orbital region with fewer than 25 cases documented. We report a case of a 60-year-old male presenting a slowly progressive, painless lower eyelid mass evolving over 10 years. Clinical examination revealed a well-circumscribed 15 mm lesion. Angio-computed tomography (CT) demonstrated a 22 × 24 × 18 mm well-circumscribed mass with peripheral contrast enhancement, consistent with a vascularized lesion. Surgical excision via a subciliary approach was performed. Histopathological analysis showed a 15 × 10 mm tumor composed of smooth muscle bundles surrounding vascular channels. Immunohistochemistry was positive for smooth muscle actin and desmin, confirming the diagnosis of angioleiomyoma. No recurrence was observed during a 3-year follow-up. The absence of magnetic resonance imaging (MRI), due to patient financial constraints, constitutes a limitation discussed herein. This case highlights the diagnostic challenge and the importance of considering angioleiomyoma in differential diagnoses of eyelid masses.

## Introduction

Angioleiomyomas are rare benign tumors composed of smooth muscle cells and thick-walled blood vessels. Although they are more frequently encountered in the lower extremities—particularly among middle-aged women—their occurrence in the head and neck region represents <10% of all cases [1]. Orbital and peri-orbital localizations are exceedingly rare, with fewer than 25 documented cases in the literature, and only a minority involving the eyelid [2, 3]. Lower eyelid angioleiomyomas are exceptionally uncommon and may clinically mimic other benign or malignant tumors.

We present a rare case of a lower eyelid angioleiomyoma in a 60-year-old male with a 10-year history of asymptomatic growth. This case underscores the diagnostic challenges and surgical considerations associated with this lesion, with an emphasis on imaging features, histopathological confirmation, and long-term clinical follow-up.

## Case report

**Figure 1:**
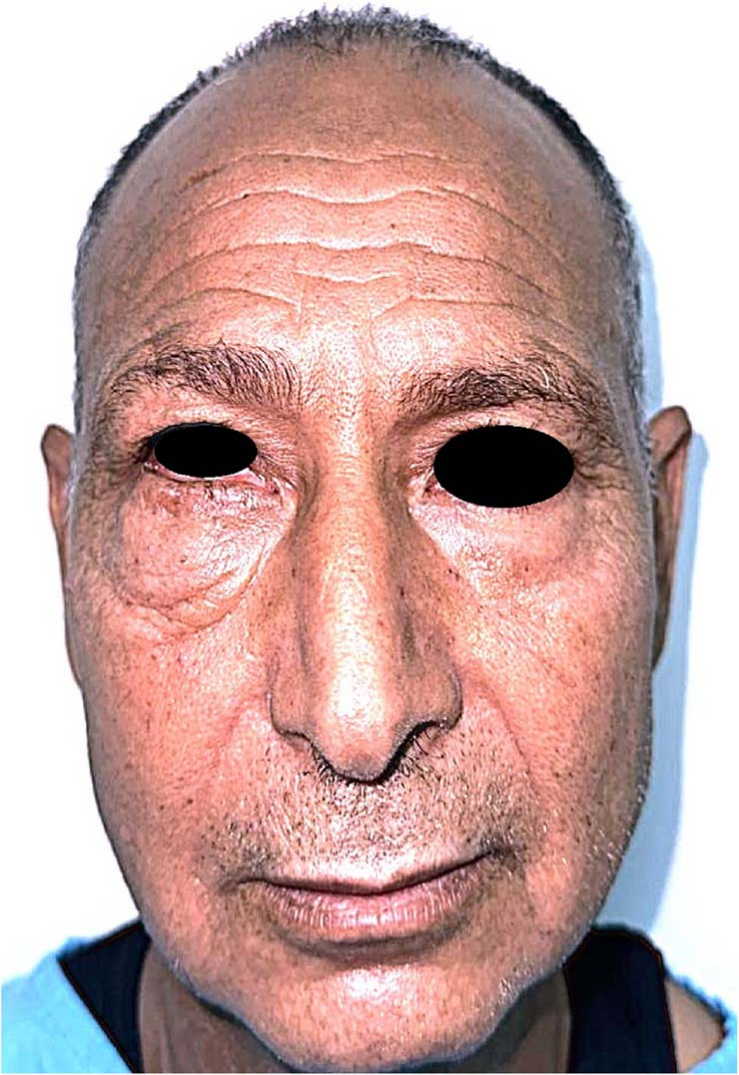
Frontal view of the patient showing a discrete, well-demarcated lower eyelid swelling without skin changes or ulceration.

**Figure 2:**
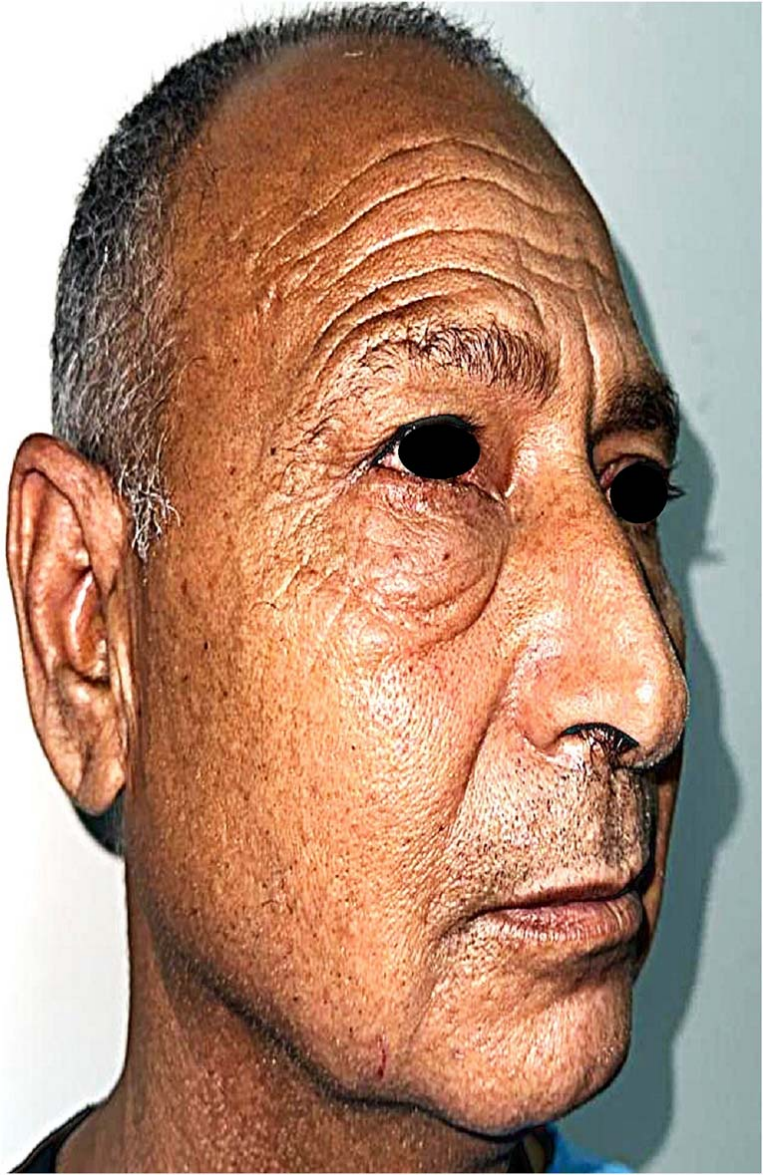
Lateral profile of the patient demonstrating the extent of the painless lower eyelid mass.

A 60-year-old man presented with a slowly enlarging mass of the right lower eyelid, evolving over 10 years without pain or functional impairment. Physical examination identified a firm, mobile, non-tender lesion measuring ~15 mm in diameter. The overlying skin was intact without ulceration or inflammation (Figs 1 and 2).

**Figure 3:**
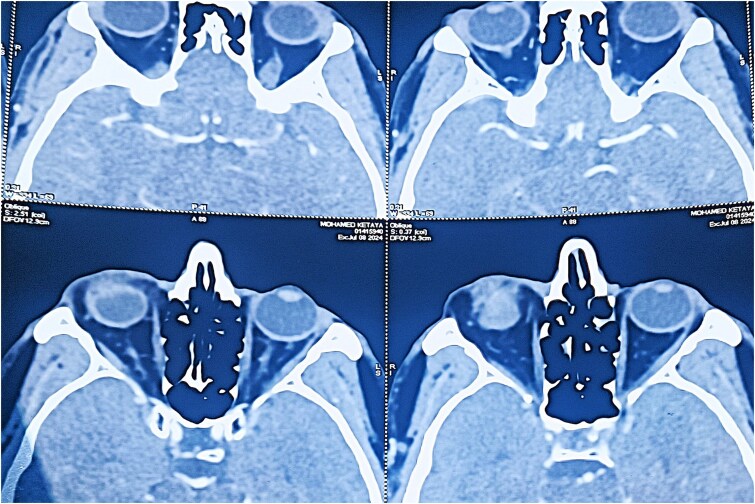
Axial CT scan (soft tissue window) revealing a well-defined, ovoid lesion with peripheral contrast enhancement in the inferior orbital region.

**Figure 4:**
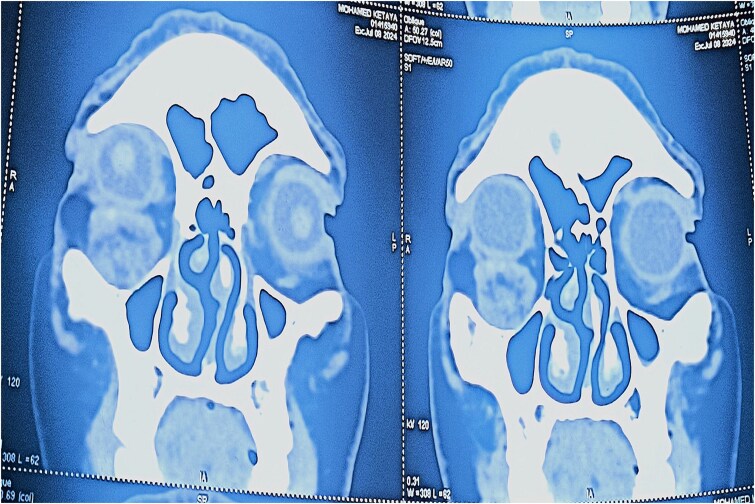
Coronal CT scan showing the subcutaneous location of the tumor with no invasion of adjacent structures.

Angio-computed tomography (CT) imaging revealed a well-circumscribed mass measuring 22 × 24 × 18 mm with peripheral contrast enhancement, consistent with a vascularized lesion. Magnetic resonance imaging (MRI) was not performed due to financial limitations. The lesion did not show signs of invasion into adjacent structures (Figs 3 and 4).

**Figure 5:**
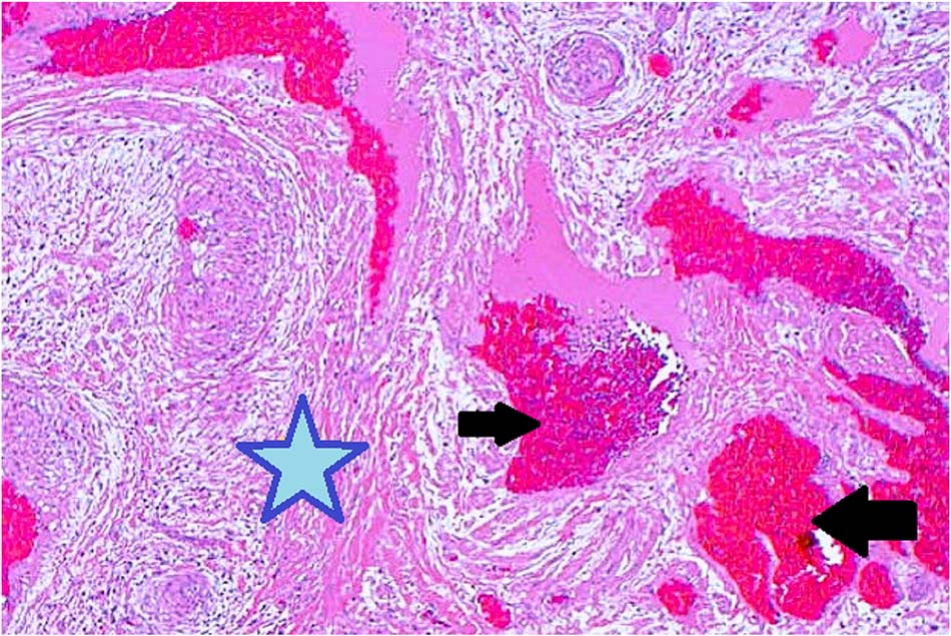
Histopathological examination with hematoxylin and eosin staining shows muscular components (marked with a star) and vascular components (marked with arrows).

Surgical excision was performed via a subciliary approach, and the mass was removed entirely. Macroscopic examination revealed a 15 × 10 mm encapsulated tumor. Histology demonstrated interlacing bundles of smooth muscle cells surrounding thick-walled vascular channels without atypia, mitotic figures, or necrosis. Immunohistochemical staining was positive for smooth muscle actin (SMA) and desmin, confirming the smooth muscle origin of the tumor (Fig. 5).

**Figure 6:**
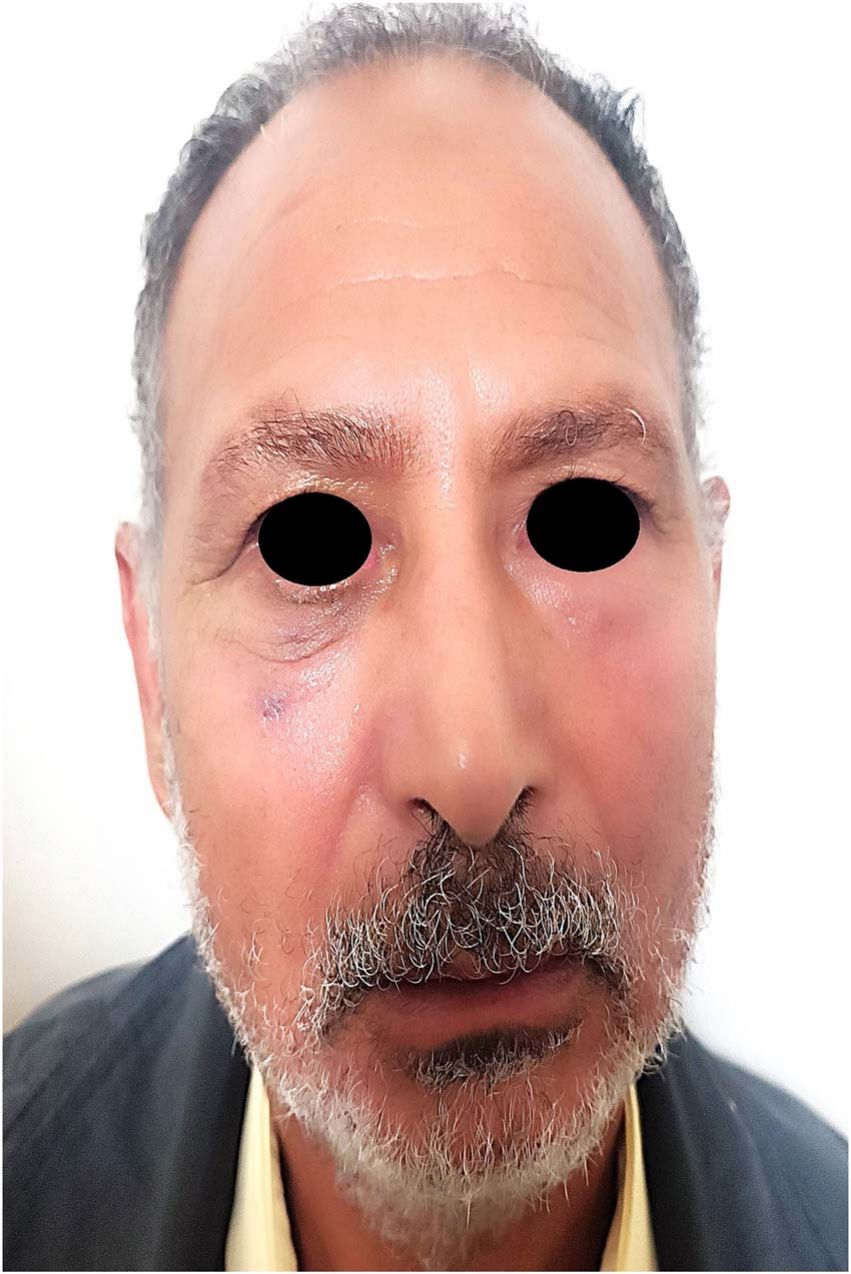
Postoperative frontal view of the patient at 1-year follow-up, showing excellent esthetic and functional outcome with no recurrence.

Postoperative recovery was uneventful, and no recurrence was observed during the 3-year clinical follow-up (Fig. 6).

## Discussion

Angioleiomyoma is a rare benign tumor arising from vascular smooth muscle cells, most commonly located in the lower extremities. Orbital and peri-orbital localizations are exceptional, with fewer than 25 cases reported in the literature [1, 2].

The lesion's clinical presentation in our case, characterized by a slow, asymptomatic growth over 10 years without functional impairment, aligns with previous reports. Unlike some cases presenting with pain or proptosis [3], our patient remained asymptomatic, emphasizing the tumor's variable clinical course.

Radiologically, the angio-CT revealed a well-circumscribed mass measuring 22 × 24 × 18 mm with peripheral contrast enhancement, typical for vascular tumors [4]. This peripheral pattern likely corresponds to the rich vascular capsule and differentiates it from other lesions exhibiting homogeneous enhancement. Unfortunately, MRI was not available for this patient due to economic constraints, representing a limitation of this report. MRI would have allowed better tissue characterization and vascular mapping, aiding preoperative planning [5].

The discrepancy between the clinical (15 mm), histopathological (15 × 10 mm), and imaging (22 × 24 × 18 mm) tumor sizes can be attributed to the peritumoral vascular and connective tissue extension captured by imaging modalities but not measured clinically or histologically. This is consistent with vascular tumor behavior, where the radiological extent may exceed the gross tumor size [6].

Histologically, the tumor showed typical features of angioleiomyoma: bundles of smooth muscle cells surrounding vascular channels without atypia or mitoses. Immunohistochemistry confirmed smooth muscle origin with positive staining for SMA and desmin. These findings exclude malignancy and other differential diagnoses such as hemangioma, schwannoma, or malignant soft tissue tumors [7].

Clinically and histologically, several features support the benign nature of the lesion: absence of ulceration, well-defined radiological margins, lack of mitotic figures or cellular atypia, and absence of local invasion. These exclude malignancies such as basal cell carcinoma or soft tissue sarcoma.

Complete surgical excision remains the treatment of choice, with low recurrence rates reported. Our 3-year postoperative follow-up without recurrence supports the favorable prognosis of angioleiomyomas in this location [8].

### Limitations

The absence of MRI imaging, due to financial constraints, limits comprehensive preoperative characterization of the lesion. Longer follow-up could further confirm the absence of recurrence. Despite these limitations, the clinical, radiological, histological, and immunohistochemical data collectively confirm the diagnosis and favorable outcome.

## Conclusion

Angioleiomyoma of the lower eyelid is an exceptionally rare entity that may remain undiagnosed for years due to its slow growth and benign appearance. Although imaging can suggest the diagnosis, definitive identification relies on histopathological and immunohistochemical analyses. Clinicians should consider angioleiomyoma in the differential diagnosis of chronic, asymptomatic eyelid masses. Early surgical excision is both diagnostic and curative. Long-term follow-up remains essential to detect potential recurrences, although they are rare.
